# Diagnosis between Infantile Remittent Fever and Hydrocephalus

**Published:** 1852-07

**Authors:** 


*Diagnosis between Infantile Remittent Fever and Hydrocephalus.* By Dr. C. Taylor.*

It is often an anxious point in infantile pathology to determine whether the cerebral symptoms which may be present in a given case, are indicative simply of sympathetic disturbance of the sensorium, or of the series of anatomical changes which characterize the disease known as hydrocephalus. In deciding the question, some assistance will be derived from the following summary of the distinctive symptoms in each:

| *Remittent Fever.* | *Hydrocephalus.* |
|---|---|
| Head, slight pain in. | Head, violent pain in; tossing of; drawn backward, and bored in pillow. |
| Delirium at night frequent; convulsions rare—sometimes at onset. | Delirium seldom; convulsions not early—more toward end of disease; aversion to light and noise. |
| Easily aroused. | Roused with difficulty; stertorous breathing; paralysis in late stage. |
| Cry fretful, if any. | Cry peculiar, sharp and shrill; frequent sighing. |
| Hands usually thrown about bed (Coley.) | Hands tossed toward head. |
| Countenance heavy and dull; vacant expression, as of fever in adult. | Countenance sometimes anxious, sometimes dull. |

---

* *Infantile Remittent fever* is to be considered as embracing *continued fever* affecting children.—Ed. Buffalo Med. Jour.

| | |
|---|---|
| Neither knitting of brows nor pupil of eye affected. | Knitting of brows; wakefulness; pupil of eye contracted in early stage — sometimes oscillatory, afterward dilated. |
| Senses of sight and hearing often dull. | Senses of sight and hearing often acute in early stage. |
| Pulse quick throughout the disease. | Pulse quick, but irregular in its action and force in early stage; often beating of carotids, and pulsation and prominence of fontanelle; pulse afterward becomes slow, but, on raising the child, again quickened. |
| Bowels occasionally constipated at first; frequently relaxed. | Bowels constipated, and very difficult to move. |
| Motions various; often clayey and deficient in bile; very offensive. | Motions peculiar and characteristic —dark green and slimy, like chopped spinach. |
| Vomiting occasionally at first, but never continuous. | Vomiting early in first stage; often very constant, especially on assuming the erect posture of sitting up. |
| Pain often in the iliac regions, particularly the right. | Pain occasionally at hypochondrium. |
| Abdomen in advanced stage sometimes tumid. | Abdomen drawn in in advanced stage. |
| Appetite mostly destroyed; will not take any thing. | Appetite sometimes good; will take food. |
| Thirst often great from commencement. | Thirst not great in first stage; often in latter stage great avidity for constant drink. |
| Tongue often loaded with yellowish white fur, in gastric form, and elongated and projected papillæ, giving it a "strawberry appearance;" red, dry, and occasionally brown, in malarial form. | Tongue white; nothing indicative. |
| Skin very hot; abdomen hotter than the head; picking of the nostrils, corners of the mouth, etc. | Skin not so hot, afterward cold; head the hottest part. |
| Paroxysms regular; exacerbations toward night, remissions in the morning. | Varies in intensity without regularity. |
| Seldom occurs under three years. | Frequent under the third year; less so after the fifth. More frequent in boys of a scrofulous habit. |

*Ranking's Abstract.*

